# Supplementary material for: Benzoxazine Containing Fluorinated Aromatic Ether Nitrile Linkage: Preparation, Curing Kinetics and Dielectric Properties
Source: Polymers (Basel). 2019 Jun 11;11(6):1036. doi: 10.3390/polym11061036 (PMC6631285; doi:10.3390/polym11061036)
Supplement: Supplementary file 1 [file polymers-11-01036-s001.pdf]

Supplementary information

Benzoxazine containing fluorinated aromatic ether nitrile linkage: Preparation, curing kinetics and dielectric properties

Sijing Chen, Dengxun Ren, Bo Li, Kui Li, Lin Chen, Mingzhen Xu*, Xiaobo Liu*
Research Branch of Advanced Functional Materials, School of Materials and Energy, University of Electronic Science and Technology of China, Chengdu 610054, China.
* Corresponding author: Tel: +86-028-83207326; Fax: +86-028-83207326; mzxu628@uestc.edu.cn , liuxb@uestc.edu.cn.

Dynamic mechanical analysis (DMA, Q800, TA Instruments, USA) was applied to study thermal mechanical properties of glass fiber (GF, EW170-100, Shenyang Gaote glass fiber Co., Ltd., Shenyang, China.) reinforced FAEN-Bz composite laminates (FAEN-Bz/GF). The FAEN-Bz/GF composites prepared as follows. 8 layers of GF were impregnated with FAEN-Bz solution. Then, the prepregs dried in oven at 80 °C for 20 min and 160 °C for 15 min to remove the solvent, and the dried prepregs were placed in stainless mold under 20 MPa at 200 °C for 4 h and 240 °C for 2 h to prepare FAEN-Bz/GF composites. DMA (three-point bending) was tested from 50 °C to 260 °C (5 °C/min) under air atmosphere with the frequency of 1 Hz. The results were presented as follows. The dimension of sample for DMA tests was 40 mm × 10mm × 0.67 mm. According to the glass transition theory, the temperature at the maximum peak of *tan* δ curve can be defined as glass transition temperature T_g. The T_g of FAEN-Bz/GF composites cured at 200 °C for 4 h and 240 °C for 2 h exhibited a high T_g (about 233 °C). Compared to the polybenzoxazine previously reported [1], polyFAEN-Bz possessed good thermal stabilities.

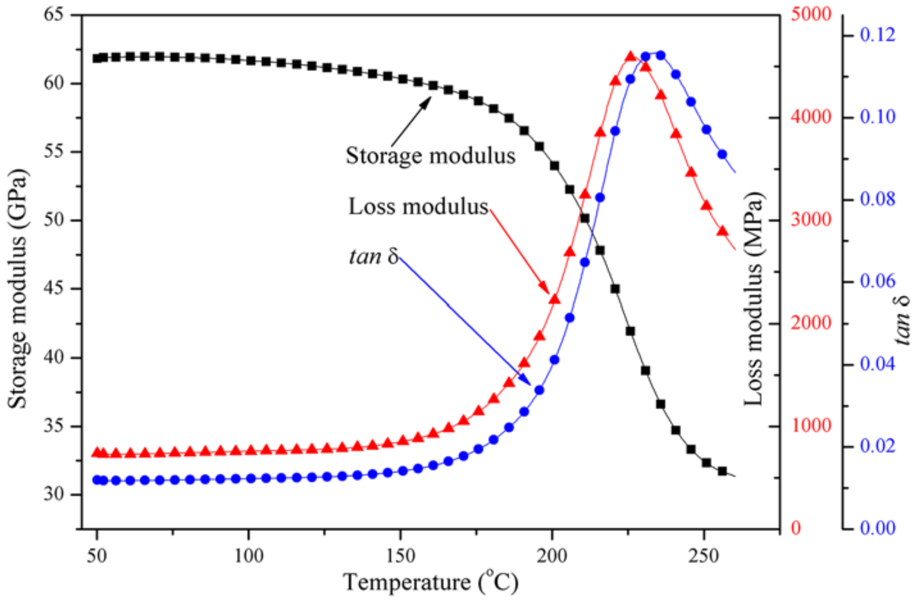

Figure S1. Storage modulus, Loss modulus and *tan* δ of DMA tests. FAEN-Bz/GF composites with curing procedure 200 °C/4 h and 240 °C/2 h.

References:

[1] Kobzar YL, Tkachenko IM, Bliznyuk VN, Shevchenko VV. Fluorinated polybenzoxazines as advanced phenolic resins for leading-edge applications. Reactive & Functional Polymers. 2018;133:71-92.
